# Roles of the Exosomes Derived From Myeloid-Derived Suppressor Cells in Tumor Immunity and Cancer Progression

**DOI:** 10.3389/fimmu.2022.817942

**Published:** 2022-01-27

**Authors:** Zhuang Chen, Rui Yuan, Shengyun Hu, Weitang Yuan, Zhenqiang Sun

**Affiliations:** Department of Colorectal Surgery, The First Affiliated Hospital of Zhengzhou University, Zhengzhou, China

**Keywords:** myeloid-derived suppressor cells, exosome, tumor immunity, cancer, immune escape

## Abstract

Tumor immunity is involved in malignant tumor progression. Myeloid-derived suppressor cells (MDSCs) play an irreplaceable role in tumor immunity. MDSCs are composed of immature myeloid cells and exhibit obvious immunomodulatory functions. Exosomes released by MDSCs (MDSCs-Exos) have similar effects to parental MDSCs in regulating tumor immunity. In this review, we provided a comprehensive description of the characteristics, functions and mechanisms of exosomes. We analyzed the immunosuppressive, angiogenesis and metastatic effects of MDSCs-Exos in different tumors through multiple perspectives. Immunotherapy targeting MDSCs-Exos has demonstrated great potential in cancers and non-cancerous diseases.

## Introduction

Malignant tumors are considered a major threat to human health (1). Studying the contribution of tumor immunity in tumor progression may improve the extremely narrow therapeutic strategy regarding cancer. Myeloid-derived suppressor cells (MDSCs) are immature cells consisting of myeloid progenitor cells, immature macrophages, immature granulocytes and immature dendritic cells. They are closely related to patients’ poor prognosis due to its powerful effects on tumor immune suppression, tumor angiogenesis, drug resistance, and tumor metastases (2–4). For example, The interaction between MDSCs and macrophages can reduce the production of IL-12 by macrophages and increase the production of IL-10 by MDSCs, which promotes tumor progression (5).

Exosomes are 40- to 100-nm small vesicles that are released by the vast majority of cells and distributed in all body fluids (6, 7). Exosomes derived from different cells perform variable functions. Exosomes carry proteins, DNA, messenger RNAs(mRNAs), noncoding RNAs(ncRNAs), and lipids (8). Exosomes exert cancer-inhibiting or cancer-promoting effects. For example, exosomal miR-19a is delivered to osteoblasts to promote bone metastasis in breast cancer (9). While human umbilical cord mesenchymal stem cell -derived exosomal miR-320a inhibits lung cancer cell growth *via* SOX4/Wnt/β-catenin axis (10). In addition, emerging studies have shown that exosomes have potential clinical applications as biomarkers for disease diagnosis and prognosis (11–14).

The exosomes derived from MDSCs (MDSCs-Exos) are involved in the function of immunosuppression, promoting tumor angiogenesis, tumor metastasis, drug resistance of malignant tumors (15–17). Here, we summarized linkages and differences between MDSCs-Exos and parental cells, as well as regulatory roles and possible diagnostic and prognostic values in tumor immunity.

## MDSCs in Tumor Immunity

MDSCs are a special kind of cells that have important tumor immunomodulatory effects, composed of several immature heterogeneous cells originating from myeloid cells (18). Normally, immature myeloid cells differentiate into mature immediately after entering the peripheral organs (19). Under a variety of pathological conditions, MDSCs expand and can be detected in blood, cancer tissue, inflammatory sites, lymph nodes and spleen (20). In the tumor microenvironment (TME), the differentiation and amplification of MDSCs is mediated by a variety of molecules, such as: granulocyte-macrophage colony–stimulating factor, granulocyte colony–stimulating factor (G-CSF), macrophage colony–stimulating factor, stem cell factor, vascular endothelial growth factor (VEGF), and polyunsaturated fatty acids (21–23). Activation of MDSCs is dependent on the following cytokines: IFN-γ, IL-1β, TNF, IL-4, IL-6, IL-13 and high mobility group box protein 1 which function through NF-κB, STAT1 and STAT6 pathways (24). According to different surface markers, MDSCs are divided into two subtypes: granulocytic MDSCs (G-MDSCs, CD11b^+^Ly6G^+^Ly6C^low^), termed polymorphonuclear MDSCs (PMN-MDSCs) simultaneously, and monocytic MDSCs (M-MDSCs, CD11b^+^Ly6G^-^Ly6C^hi^) (23, 25, 26). The number of G-MDSCs is far outweighed by that of M-MDSCs, and a majority of current studies have focused on investigating the capabilities of G-MDSCs, while the role of M-MDSCs remains to be further investigated (27). Their immunosuppressive abilities and mechanisms also differ (23, 28). G-MDSCs inhibit T-cell responses mainly through the production of reactive oxygen species (ROS) by antigen-specific methods. M-MDSCs produce large amounts of NO, arginase 1 (Arg-1) and immunosuppressive cytokines, such as IL-10, which inhibit both antigen-specific and non-specific T-cell responses. M-MDSCs have a higher inhibitory activity than G-MDSCs (29–33).

MDSCs exert their immunosuppressive effect by promoting the expansion of regulatory T cells (Tregs) (34–36), promoting the production of helper T cell 17 (Th17) (35), inducing macrophage differentiation into the M2 phenotype (2, 37, 38) and inhibiting of immune response of NK cells (39) and B cells (40). In addition to suppressing the immune response, MDSCs also accelerate tumor progression by remodeling tumor microenvironment (22). MDSCs promote tumor angiogenesis through up-regulating VEGF, matrix metallopeptidase 9 (MMP9) and bombina variegata peptide 8 (Bv8) (41). MDSCs also promote tumor metastases by infiltrating primary tumors and facilitating the formation of premetastatic niches (42).

In conclusion, MDSCs exhibit great prospect in the treatment of cancer by blocking T cells, B cells and NK cells activity and bolstering Tregs expansion and mobilization.

## Exosome

Extracellular vesicles (EVs) were initially considered to be “platelet dust” by Peter Wolf in 1967 (43). EVs transport proteins, nucleic acids, lipids, cytokines, metabolites, and enable intercellular information communication (44). Depending on their origin, markers, properties and functions, EVs are classified into two main groups: ectosomes and exosomes (45). Ectosomes are vesicles formed by outward budding of the plasma membrane, whereas exosomes are intraluminal vesicles formed by plasma membrane invagination, the release of the latter involves the fusion of multivesicular bodies with the plasma membrane (46–48). Recent studies suggest that CD63 is the signature exosome-specific protein, while CD9 and CD81 are not specific (49). Statistically, the cargoes that have been identified in exosomes include 9769 proteins, 3408 mRNAs, 2838 miRNAs and 1116 lipids [data from http://www.exocarta.org (a database collecting many studies)]. These components are involved in cellular signaling pathways, regulation of lipid metabolism, tumor progression, recurrence and metastasis (50–53).

After exosomes are released outside the cell, they participate in information transmission with the target cells through membrane fusion, endocytosis and binding to the receptors on the surface of the target cells (54). Currently, it is becoming increasingly evident that exosomes play an essential role in disease, especially in tumors by promoting the reprogramming of receptor cells (55–57). In non-small cell lung cancer(NSCLC), tumor-derived exosomes(TDEs) polarize macrophages to an immunosuppressive phenotype that increases programmed death ligand-1 expression through NF-kB-dependent, glycolysis-dominated metabolic reprogramming, triggering the formation of pre-metastatic niche (58). Exosomal lncARSR propagates sunitinib resistance through competitive binding of miR-34/miR-449 in renal cell carcinoma (59).

In briefly, exosomes participate in the physiopathological processes of coagulation, inflammation, angiogenesis and immune response (12). Exosomes are widely distributed and easy to modulate, can be used as a promising minimally invasive tool for diagnosis and treatment (7, 60–62).

## Characterization of Exosomes Derived From MDSCs

It is now known that exosomes carry proteins, DNA, messenger RNAs(mRNAs), noncoding RNAs(ncRNAs), and lipids. MDSCs-Exos exert a unique function due to the specificity of the cargoes carried. MDSCs-Exos are also rich in proteins, RNA and DNA. We next discuss the properties of exosomes in five dimensions.

### Protein Differences Between MDSCs and Their Exosomes

It is well known that protein is the material basis of life activities (63). The same protein exists in different structures and performs different biological functions due to post-translational modifications (64). Current studies have shown that the cargoes carried by MDSCs-Exos are mainly involved in the immunosuppressive effect of MDSCs (65). S100A8/A9 (calcium binding protein, with chemotactic activity) is present in both MDCSs and MDSC-EXO. Chronic inflammation increased S100A8/A9 content in MDSCs (66), with insignificant changes in exosomes (65). At present, numerous studies on exosomes in tumor-bearing mice are mainly focused on the differences of cargoes (especially ubiquitination protein (67), glycoprotein (68, 69) and RNA (70, 71) carried by exosomes and parental cells.

A study identified 1726 proteins in MDSCs and their exosomes, of which 58% were identified in MDSCs and their exosomes simultaneously. Regardless of inflammation, 30% of the proteins in MDSCs are enriched in their exosomes, especially those involved in exosome formation and protein sorting as well as proteins that load miRNAs into exosomes. Through this selective sorting mechanism, MDSCs-Exos may mediate some functions different from those of MDSCs (15). Similar to other exosomes, MDSCs-Exos enrich many characteristic components, such as tetraspanins (including CD9, CD177), Hsp70, Hsp90α, Hsp90β, Alix, and the ESCRT complex, which are involved in exosome formation and protein sorting. Compared with parental cells, the abundance of CD9 was 89-fold increased regardless of inflammatory status (15, 65). MDSCs-Exos also contain many other protein cargoes, including many nucleic acid binding proteins, numerous histone variants and several elongation factors. It has been reported that these proteins can bind to nucleic acids and induce changes in nucleic acids expression and the protein spectrum of receptor cells (72). Some chemotactic proteins are enriched in MDSCs-Exos, such as the pro-inflammatory proteins S100A8/9, CD47 and thrombospondin-1. These proteins mediate the aggregation of MDSCs and enhance the immunosuppressive function of MDSCs. The relative abundance of the pro-inflammatory proteins S100A8/9, which are secreted by MDSCs and mediate >90% of the chemotactic effect on MDSCs, are not affected by inflammatory conditions. The cytokine macrophage migration inhibition factor and the chemokine platelet factor-4 are also enriched in exosomes, and these proteins exhibit chemotactic activity on leukocytes (15). Regardless of inflammatory conditions, transforming growth factor-β1(TGF-β1) is 4.3-fold more abundant in the exosomes compared with parental cells (15). TGF-β1 participates in the expansion of T cells and the inhibition of NK cells (73, 74). Immunoglobulins, complement regulatory factor H and C4B-binding proteins(C4B-bp) are enriched in exosomes. They may also be involved in the regulation of immune system by MDSCs and their exosomes (15).

The above results indicated that the cancer-promoting effects of MDCSs are partially achieved by exosomes. Targeting MDSCs-Exos holds a bright future for cancer treatment.

### Differences Roles of the Exosomes Released by G-MDSCs and M-MDSCs in Tumor

As we mentioned earlier, MDSCs are distinguished into two subtypes. Interestingly, the exosomes derived from different subtypes of MDSCs also differ in their impacts on tumor. Rab27a controls exosomes biogenesis (75). The expression of Rab27a was significantly reduced by transfecting siRNA. In a tumor sphere formation assay, after inhibiting of exosome derived from G-MDSCs, the tumor sphere numbers, CD44+ cell percentages and CD133+ cell percentages were decreased. But the CD44+ cell percentages was not decreased when exosomes were inhibited in M-MDSCs (76). This indicated that G-MDSCs-Exos and M-MDSCs-Exos have different effects on cancer cell stemness.

Currently, researchers have mainly focused on investigating the role of G-MDSCs-Exos on tumor progression, and studies on M-MDSCs-Exos are very rare. Although there is an evidence that M-MDSCs-Exos affects tumor immunity. However, researchers have mainly focused on investigating the role of G-MDSCs-Exos on tumor progression, and studies on M-MDSCs-Exos are very rare. To some extent, an insight into the role of M-MDSCs-Exos may lead to new immunotherapeutic approaches. Distinguishing the role differences between M-MDSCs-Exos and G-MDSCs-Exos may be a new research hotspot.

### Differences in Proteins Carried by MDSCs-Exos Under Different Inflammatory Conditions

As inflammation increases, an increasing number of MDSCs were identified, and stronger immunosuppressive effect was observed (77). MDSCs play a key role in the control of experimental necrotizing small intestinal colitis in neonatal mice by suppressing T-cell function (78). Immunosuppressive proteins and miRNAs are increased in EVs during chronic inflammation and aging (79). In one study, the researchers identified 412 proteins, of which the abundance of 63 proteins changed greater than 2-fold in an inflammatory environment. It is worth noting that there was no obvious difference in quantity of exosomes shed per MDSC isolated from low-inflammation or high-inflammation environments. Compared with conventional conditions, inflammatory conditions reduced the abundance of 33 proteins, such as C4B-bp, complement C3 and ficolin-1, which participate in the innate immune response. Several cytoskeletal proteins and chemotactic proteins are found to be reduced in an inflammatory environment, which are related to the migration of exosomes. In addition, a highly inflammatory environment increased the abundance of 30 proteins, including Leukocyte elastase inhibitor A, DBF4-type zinc finger-containing protein 2 homologue and Cathepsin G, etc (65).

Reducing inflammation may expand new horizons for cancer treatment by weakening MDCSs in TME. In the future, the majority of cancer patients may be able to benefit from this.

### Ubiquitin Proteins and Glycoproteins Carried by MDSCs-Exos

Ubiquitin is a common post-translational modification (80), which affects protein function by influencing protein stability, turnover, cellular localization, and regulating cellular signaling cascade responses (81). The imbalance between ubiquitination and deubiquitination is closely related to the occurrence of human immune diseases, cancer, infection and neuropathy (82). In NSCLC, deubiquitination of PDL-1 promotes immune escape by suppressing CD8^+^T cell responses (83). NLRC3, a member of the innate immune receptor, impaired CD4^+^ T cell signaling and metabolism by limiting NF-κB activation, reducing glycolysis and oxidative phosphorylation *via* decreased K63-linked ubiquitination of TNF-receptor-associated factor 6 (84). Therefore, it is urgently needed to investigate whether MDCSs and MDSCs-Exos carry ubiquitin protein, which will help to develop new treatment strategies based on exosomes. Protein blot analysis demonstrated that the parental cells and their exosomes contained different ubiquitinated protein profiles (85). Initially, 10 ubiquitinated proteins in MDSCs-Exos were identified (65). With the application of mass spectrometry-based bottom-up proteomics technology, scholars isolated and identified 50 ubiquitinated proteins from MDSCs-Exos (86). Specifically, the ubiquitinated nuclear proteins include several histones, ribosomal proteins and nucleic acid binding proteins. The ubiquitinated histones in these exosomes may possess active pro-inflammatory properties (87, 88). Interestingly, the pro-inflammatory high mobility group box protein 1 is ubiquitinated, promotes the accumulation of MDSCs, and enhances the immunosuppressive effect of MDSCs (89). Sorting nexin 13 has been identified to be involved in endosomal transport of ubiquitinated proteins (90). Two ubiquitinated keratins were revealed to play an active role in plasma membrane invagination during the initial phase of EVs formation (91, 92). Other ubiquitinated proteins leucine zipper EF hand-containing transmembrane protein 1 and endoplasmin, which participate in the formation of endosomes and exosomes (65).

Similarly, glycosylation is an important protein modification that determines protein folding and transport and is crucial for mammalian survival (93, 94). Until 2018, 21 N-glycoproteins on the surface of MDCS-Exos exosomes were identified using proteomic methods, including CD44, CD47, CD321, CD157, CD11b, CD97, thrombospondin1 (Tsp1), fibronectin, cytoskeletal krt, fibrinogen, etc (95). Of special interest is CD47, donor CD47 plays an important role in the control of T cell allogeneic response and tolerance induction after hepatocyte transplantation (96, 97). It mediates the chemotaxis and migration of MDSCs by combining with Tsp1 on MDSCs. When CD47 on tumor cells binds to CD172a (signal regulatory protein α or SIRP α), it can prevent macrophages from phagocytosing tumors (98) and maintain acquired immune tolerance (97). Therefore, CD47 is a potential drug target (99). In addition, it is worth noting that MDSCs-Exos may transport immunosuppressive cargoes to T cells through the binding of CD321 to TLFA-1 of T cells (95).

Both ubiquitinated and glycosylated proteins are present in MDSC and its derived exosomes, respectively, which supports the idea that exosomes have an analog to parental cells.

### mRNAs and miRNAs Carried by MDSCs-Exos

In addition to carrying protein cargo, MDSCs-Exos also carry a large number of RNAs, including mRNAs and miRNAs (15), similar to the results of previous studies, almost no ribosomal RNA was found (100–102). The mRNAs carried by exosomes is also transferred to recipient cells and translated into functionally active proteins, which produces more lasting effects than proteins. Compared with parental cells, 45% of mRNA transcripts in exosomes exhibited statistically differences in abundance regardless of the inflammatory conditions. The transcripts of these mRNAs took part in several signaling pathways including “calcium signaling pathway”, “cAMP signaling pathway” and “hippo signaling pathway”. In addition, only approximately 3.5% of the mRNA transcripts differ in abundance under inflammatory conditions compared with parental cells. These mRNA transcripts played role in the signaling pathway associated with TGF-β and VEGF. Compared with conventional exosomes, several biological processes were identified enriched in inflammatory exosomes, including “cell-cell signaling”, “macrophage differentiation” (15).

Simultaneously, the study identified approximately 1500 differentially expressed miRNAs in MDSCs-Exos, and approximately half of them exhibited increased abundance in inflammatory exosomes. According to the prediction of these miRNA targets, if these miRNAs are transferred to the target cells and bind to mRNA targets, they will affect the proliferation, differentiation and apoptosis of target cells. These miRNAs can regulate the immune system and tumor microenvironment and thus affect tumor progression and metastasis.

The miRNAs enriched in inflammatory exosomes include miRNA-704, miRNA-5134, miRNA-7022 and miRNA-7062, which bind to the target mRNA taking part in the apoptosis pathway, including Fas. Compared to parental cells, miRNA-690 and miRNA-155 are enriched in exosomes and may be delivered to MDSCs. MiRNA-690 promotes MDSCs expansion through regulating the cell cycle of myeloid cells. MiRNA-155 increases the production of IL-10. IL-10 induces the proliferation of regulatory T cells and causes the transformation of macrophages to tumor growth-promoting M2-Mϕ. Interestingly, miR-146a negatively regulates the activation of the NF-κB pathway and subsequently controls inflammation by targeting the IL-1 receptor-associated kinase 1 and TNF receptor-associated factor 6 mRNAs (15, 103). In contrast to miRNA-690 and miRNA-155, miR-146a suppresses the development of malignant tumors (15).

We have realized that MDSCs-Exos regulate the signaling pathways and biological processes of target cells through the carried proteins and RNAs. At present, it is necessary to further clarify the type and abundance of cargoes contained in MDSCs and their exosomes, and compare the similarities and differences of cargoes carried by MDSCs and exosomes under different conditions. It is helpful to predict the function of MDSCs-Exos according to the existing research on the composition and function of MDSCs.

## Effects of MDSCs-Exos on Tumor Immunity in Various Cancers

Tumor immunosuppression is a feature of malignant tumors (104). MDSCs-Exos play an irreplaceable role in tumor immunity, similar to parental MDSCs. Here, we summarized the role of MDSCs-Exos in cancer immunity.

### Immune Suppression Induced by MDSCs-Exos in Cancers

MDSCs are one of the components of TME and are involved in tumor progression mainly by suppressing the function of T cells (26). MDSCs-Exos, as the immunosuppressive factor in the TME, carry many bioactive substances from MDCSs. In tumor-bearing mice, MDSCs-Exos were significantly higher in tumor tissue than at the spleen and bone marrow. MDSCs-Exos activate CD8+ T cells and drive them to produce more IFN-γ, but MDSCs-Exos increase ROS production, activate the Fas/FasL pathway in T cells, and trigger so-called activation-induced cell death (AICD) (105, 106). In tumor patients, this process is induced by the high expression of S100A8/9 (20). TDEs-provided membrane-associated Hsp72 triggers the activation of TLR2/MyD88-dependent STAT3 pathway in MDSCs through autocrine IL-6, which triggers significant immunosuppressive activity (107). The miRNAs carried by TDEs are also involved in enhancing the expansion and immunosuppression of MDSCs. For example, hypoxia-inducible miRNA-21 in TDEs enhances MDSC expansion and activation by targeting RORα and PTEN (108).

MDSCs-Exos regulate tumor immunity by carrying differential bioactive contents that mainly act on MDSCs and other target cells in the immune system. The pro-inflammatory S100A8/9 heterodimer carried by MDSCs-Exos is chemotactic for MDSCs and plays the primary role in promoting the aggregation of MDSCs to the tumor tissue and pre-metastatic niche (109). Other chemotactic proteins enriched in MDSCs-Exos include CD47 and TSP1, which mediate the immunosuppressive function of MDSCs together with S100A8/9 (65). Similar to MDSCs, MDSCs-Exos can also transform macrophages into tumor growth-promoting M2 macrophages by reducing the production of IL12 from macrophages (65). TGF-β1 enriched in MDSCs-Exos induces Tregs or Th17 cells and impair the cytotoxicity of natural killer (NK) cells, which enhances the immunosuppressive effect of MDSCs-Exos (15).

### Contribution of MDSCs-Exos to Tumor Progression and Metastasis

MDSCs and their exosomes participate in the entire process of tumor progression through immunosuppression, angiogenesis, invasion and metastasis, the formation of a premetastatic niche and the stemness of tumor cells. G-MDSCs gathered in lung cancer tissue secreted a large amount of miRNA-143-3p. MiRNA-143-3p promotes tumor cell proliferation by inhibiting integral membrane protein 2B and activating PI3K/Akt pathway (110).

Angiogenesis is fundamental for the growth and metastasis of solid tumors (111). Tumors can induce the upregulation of growth factors, including VEGF, ANG, PDGF, TGF and EGF, which disrupt the balance between proangiogenic and antiangiogenic signals. Growth factors also induce the ‘‘angiogenic switch’’ and subsequently promote the proliferation of vascular endothelial cells and the formation of capillaries (112). In addition, hypoxia in the tumor microenvironment aggravates this process by increasing the expression of proangiogenic factors (113). Several recent studies demonstrated that MDSCs and their exosomes also participate in tumor angiogenesis by recruiting MDSCs to the tumor site with several chemokines. MDSCs can secrete proangiogenic factors, including BV8 (bombina variegata peptide 8), VEGF, and basic fibroblast growth factor, by activating the STAT3 signaling pathway (41). MDSCs can also produce MMP-9, a protease that degrades extracellular matrix, which triggers the release of VEGF deposited in the matrix and increases its bioavailability (114). Moreover, the production of CCL2 in the TME is another important mechanism by which MDSCs promote tumor angiogenesis (115). Notably, splenic MDSCs can differentiate into endothelial progenitor cells that directly participate in tumor angiogenesis (116). It has been reported that miR-126a+MDSCs induced by doxorubicin (DOX) treatment in breast tumor-bearing mice interact with IL-13+Th2 cells in a positive feedback loop manner, increasing the production of Th2 cells and miR-126a+MDSC-Exo. Consequently, the increased level of miR-126a+MDSC-Exo lead to lung metastasis by promoting tumor angiogenesis (17).

**Figure 1:**
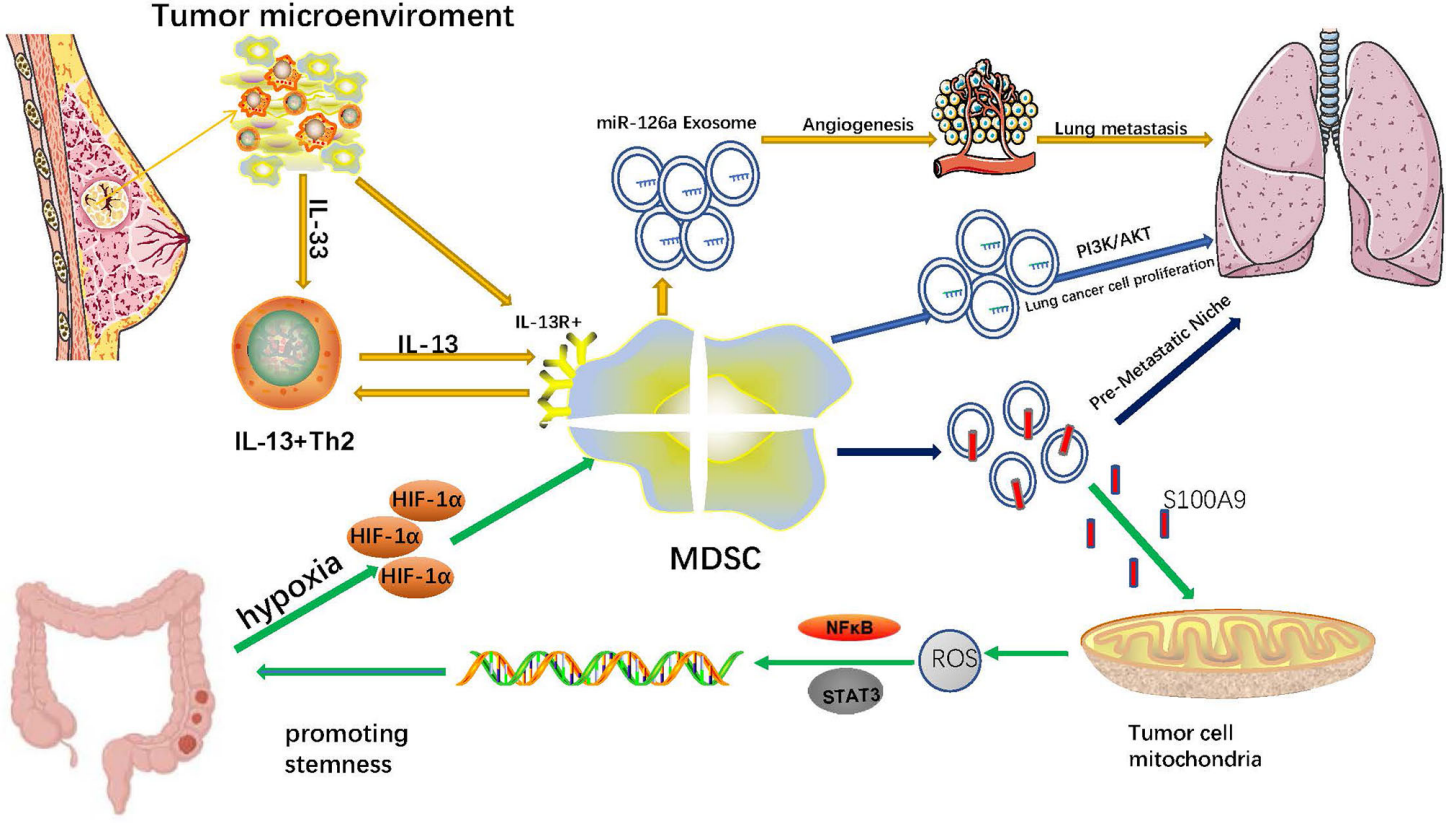
Immunoregulator effects of MDSCs-Exos in cancers.

One of the negative features of malignant tumors is their unlimited proliferation ability, and cancer stem cells (CSCs) endow them with this ability (117). CSCs have been considered a significant supporter of tumor progression and chemoresistance, and emerging evidence suggests that MDSCs and their exosomes exert crucial influence on the stemness of tumor cells. In patients with ovarian cancer, MDSCs induce ovarian cancer cells to express microRNA101. MicroRNA101 increases the expression of stem cell genes, including OCT3/4, SOX2, and NANOG, *via* inhibiting the expression of C-terminal binding protein-2 in ovarian cancer cells. As a result, MDSCs promote the stemness of ovarian cancer cells (118). In breast cancer patients, MDSCs promote cancer cell stemness by activating the NO/NOTCH and IL-6/STAT3 signaling pathways (119). New evidence in cervical cancer patients indicates that MDSCs induced by tumor-derived G-CSF enhance the stemness of cancer cells *via* producing Prostaglandin E2(PGE2) (120). MDSCs infiltrate into PTEN null prostate cancer cells and induce the stemness of prostate cancer cells *via* producing IL-1Ra and blocking the IL-1α/IL-1R axis (121). In addition, MDSCs promote the stemness and induce mesenchymal characteristics of pancreatic cancer cells by upregulating the levels of p-STAT3 (122). In A549 transplantation tumors treated with endostatin, MDSCs and MDSC-derived TGF-β1 and hypoxia enhanced the stemness of A549 cells and their resistance to endostatin (123). A new study of colorectal cancer revealed that hypoxia can promote G-MDSCs to generate more MDSCs-Exos by up-regulating HIF-1α. MDSCs-Exos aggravate the stemness of colorectal cancer cells through exosomal S100A9. Blocking S100A9 expression in MDSCs-Exos can inhibit the stemness of colorectal cancer cells and prevent the occurrence of colon cancer in mice with colitis (76) (**Figure 1**).

### The Role of MDSCs in Tumor Chemoresistance

Chemotherapy is one of the most important treatments for malignant tumors, but chemoresistance is a crucial obstacle impeding clinical treatment (124). At present, the mechanism underlying chemoresistance has not been well elucidated, but several emerging lines of evidence suggest that MDSCs induce reduced tumor cell sensitivity to chemotherapy. In mice with colorectal cancer, oxaliplatin leads to chemoresistance by restraining the polarization of MDSCs into M1-like macrophages. In addition, MDSCs and their differentiated M2-like macrophages promote immunosuppression, angiogenesis and chemoresistance by producing protumorigenic cytokines (IL-10, TGF-β, VEGF and proteases) and suppressping the function of CD8+ T cells (125).

Tumor-derived G-CSF increases the production of MDSCs and attenuates the spontaneous apoptosis of MDSCs by activating the STAT3 pathway. Then, the increased G-MDSCs induce angiogenesis through Bv8, which leads to chemoresistance in cervical cancer. The study also showed that treating mice with depleting MDSCs enhances the effect of chemotherapy for cervical cancer (126). It has been reported that IL-6 released by drug-resistant hepatocellular cancer promotes the expansion and activity of MDSCs, and the interactions between IL-6 and MDSCs promote the chemoresistance of hepatocellular cancer. The sensitivity to chemotherapy can be enhanced *via* depleting MDSCs or blocking IL-6 (127). Similarly, PMN-MDSCs promote multiple myeloma survival in response to chemotherapies, such as doxorubicin and melphalan, and the process is mediated by soluble factors, including IL-6 (128). Benzyl butyl phthalate exposure aggravates the resistance of breast cancer to doxorubicin. Mechanically, it promotes MDSCs to infiltrate into tumors and increases the secretion of S100A8/A9 by MDSCs (129). A study shows that MDSCs promote Tregs infiltrate into lung tumors and trigger CD8T cells depletion, which strongly induces immunosuppression and chemoresistance (130).

In numerous studies, MDSCs-Exos exhibit a highly similar role to MDSCs, but whether they play a role in tumor drug resistance has not been explored. In-depth studies on the role of MDSCs-Exos in drug resistance may provide new perspectives for anti-cancer treatment strategies.

## Effects of MDSCs-Exo in Non-Oncologic Diseases

**Figure 2:**
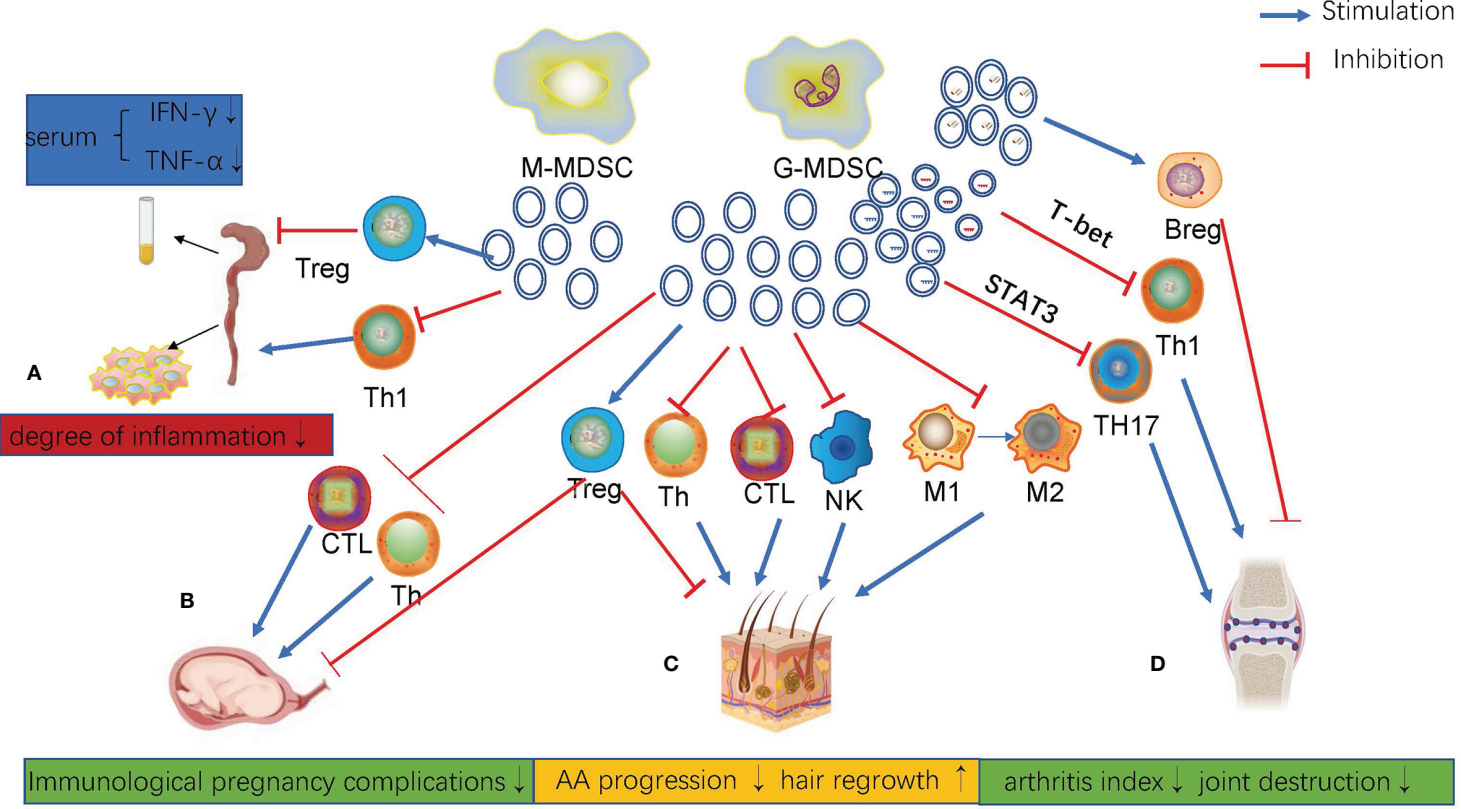
**(A)** In DSS-induced colitis, G-MDSCs-Exos inhibited the proliferation of Th1 cells, promoted the proliferation of Treg cells, and decreased serum IFN-g and TNF-AIN levels in mice. **(B)** Maternal peripheral blood G-MDSCs-Exos regulated different subtypes of T cell differentiation and function. **(C)** MDSCs-Exos inhibited AA progression and promoted hair regrowth. **(D)** G-MDSCs-Exos attenuated CIA in mice by regulating Th1, Th17 and Breg cells.

Except in tumors, the massive expansion of MDSCs is always accompanied by non-oncologic diseases, especially in autoimmune diseases. Unlike their role in tumors, MDSCs and MDSCs-Exos can alleviate autoimmune diseases (131). Exosomes derived from MDSCs carry a variety of bioactive contents, including proteins and RNAs, which play a similar role as MDSCs. MDSCs play an irreplaceable role in maternal-fetal tolerance in normal pregnancy. The isolation and identification of exosomes from maternal peripheral blood G-MDSCs revealed that G-MDSCs-Exos inhibited CD4^+^T cells and CD8^+^T cells, induced Tregs production, Th2 cell differentiation, and this effect was preserved under frozen conditions (132). (**Figure 2B**). This is very beneficial for application in clinical treatment. G-MDSCs-Exos attenuated the damage of inflammatory cell infiltration and reduced the activity index of DSS-induced colitis in mice, thus significantly alleviating the severity of the disease. This effect was mainly achieved *via* repressing the proliferation of Th1 cells, promoting the expansion of Tregs, and reducing the levels of serum IFN-γ and TNF-α in mice (133) (**Figure 2A**). In the mouse model of autoimmune alopecia areata (AA), MDSCs-Exos reversed the progression of the disease and promoted hair regeneration. MDSCs-Exos accumulated in the draining lymph nodes and cells near residual hair follicles. They are absorbed by T cells, macrophages and NK cells, especially Tregs. As a result, MDSCs-Exos significantly alleviated the disease by amplifying Tregs, weakening the cytotoxic activity of T cells, reducing the proliferation of T helper cells and increasing lymphocyte apoptosis (134) (**Figure 2C**). In mice with collagen-induced arthritis, G-MDSC-derived exosomes attenuated joint destruction efficiently by reducing the number of Th1 and Th17 cells. Mechanistically, miR-29a-3p carried by G-MDSCs-Exos targets T-bet to suppress the differentiation of Th1 cells, and miR-93-5p carried by G-MDSCs-Exos targets STAT3 to suppress the differentiation of Th17 cells (16). There is no such ability in M-MDSCs exosomes. Under hypoxic conditions, the higher levels of miR-29a-3p and miR-93-5p in G-MDSCs-Exos more effectively inhibited the proliferation of CD4^+^T cells and thus more effectively attenuate arthropathy (135). In addition, PGE2 in MDSCs-Exos upregulated the phosphorylation levels of GSK-3β and CREB to promote IL-10+ Breg cell production to attenuate CIA in mice. This effect was blocked by celecoxib (136). (**Figure 2D**)

Although the understanding of the relationship between MDSCs and autoimmune diseases has become increasingly clear, research on the role of MDSCs-Exos in autoimmune diseases remains limited. Because abundant bioactive molecules overlap in MDSCs and MDSCs-Exos, the function of MDSCs-Exos can be predicted and verified according to known research on MDSCs. Related researches will provide fresh insight into the diagnosis and treatment of autoimmune diseases.

## Clinical Applications of MDSCs-Exos

Given the crucial role of MDSC-mediated immunosuppression in tumor progression, several studies have explored a number of therapeutic strategies by targeting MDSCs. These treatments mainly include two aspects. On one hand, the number of MDSCs are reduced by using chemotherapeutic drugs (137), inhibiting the expansion of MDSCs (138) and promoting the differentiation of myeloid cells (139). On the other hand, the functions of MDSCs are suppressed. For example, nitroaspirin is used as an ROS inhibitor (140), and cyclooxygenase 2 inhibitors are used to restrain the production of arginase 1 (141). In addition, phosphodiesterase 5 inhibitors are used to suppress the production of iNOS (142).

In recent years, research on the clinical application of exosomes has become a hot topic. In terms of diagnosis, proteins and ncRNAs expressed in exosomes can be used as markers for early diagnosis, drug sensitivity and prognosis of many cancers (143). In terms of treatment, exosomes are used in tumor immunotherapy and as a new carrier for loading drugs, proteins and ncRNAs (144). When exosomes contact with the extracellular matrix or membrane of the target cells, the exosome contents will be directly transported into the target cells. According to these phenomena, drugs can be loaded into exosomes to target specific areas to treat the disease. At present, there are only a few attempts on the clinical application of MDSCs-Exos. The level of plasma S100A9 expressed in exosomes in patients with colorectal cancer is significantly increased compared with that in normal controls, and the serum level in patients with recurrent tumors is increased compared with that in patients with successful resection of colorectal cancer. Consequently, MDSC-Exo S100A9 can be used as a marker to predict the occurrence and development of colorectal cancer (76). In addition, respiratory hyperoxia inhibits the stemness of colorectal cancer cells by reducing the production of G-MDSCs-Exos, which may be used to assist in the treatment of colorectal cancer. Similarly, breast cancer patients who are resistant to DOX chemotherapy exhibit high levels of circulating miR-126a+MDSCs-Exos in their serum. Therefore, miR-126a+MDSC-Exo can be used as a potential biomarker of chemotherapy resistance to DOX in breast cancer and to guide the use of DOX in the treatment of breast cancer patients. Moreover, the systematic application of miR-126a inhibitor can improve the chemotherapeutic efficiency of DOX against lung metastasis by inhibiting tumor angiogenesis, which provides a basis for targeting MDSCs-Exos (17).

Non-steroidal anti-inflammatory drugs, glucocorticoids, and immunosuppressants have always been the main treatments of autoimmune diseases, and now MDSCs-Exos may be used as a new treatment strategy. Exosomes derived from G-MDSCs relieved collagen-induced arthritis by inhibiting the proliferation of Th1 and Th17 cells (16) and inducing IL10^+^Breg cells (136). Furthermore, high expression of miR-29a-3p and miR-93-5p induced by hypoxia in exosomes improved the condition (135). In addition, the application of G-MDSC-derived exosomes attenuated DSS-induced colitis by decreasing the percentages of Th1 cells and promoting the expansion of Tregs (133). MDSCs-Exos were also used in the treatment of autoimmune alopecia areata, which reversed the progression of the disease and promoted hair regeneration (134). Relay transfer of MDSCs protected pregnant mice from miscarriage, and exosomes exhibited similar effects, making MDSCs-Exos a possible target for the treatment of immune pregnancy complications. However, its safety as well as reliability still need to be further explored in depth (132).

There are still many blank areas in related research, and it is a demanding task to gain insight. However, future research on the characteristics, mechanism and clinical application of MDSCs-Exos will offer promising information.

## Conclusion

In summary, MDSCs-Exos play multiple roles in the tumor immunity. MDSCs-Exos exhibit tumor immunosuppressive, angiogenic and metastatic effects similar to parental cells due to similar substances to parental cells. However, research on functions, mechanisms, and contribution rates of MDSCs-Exos in tumor immunity is limited. Thus, the scientific questions that require urgent exploration include: 1. What other specific substances in MDSCs-Exos are highly effective in affecting tumor immunity? 2. Whether these substances regulate each other and subsequently affect tumor immunity? 3. How to apply MDSCs-Exos to advanced cancer patients as soon as possible?4. How to ensure the safety of MDSCs-Exos in clinical applications?

To conclude, MDSCs-Exos play an essential role in the tumor immunity. Further investigation of MDSCs-Exos in tumor immunity will be beneficial for overcoming tumor progression, recurrence, metastasis and drug resistance, providing potential biomarkers and targets for immunotherapy of cancers. Therefore, people no longer turn pale at the mention of a “cancer” in the future.

## Author Contributions

ZS and WY provided direction and guidance throughout the preparation of this manuscript. ZC and RY wrote and edited the manuscript. SH reviewed and made significant revisions to the manuscript. All authors read and approved the final manuscript.

## Funding

This study was supported by The National Natural Science Foundation of China (82173055, 81972663, U2004112), Key Scientific Research Projects of Institutions of Higher Education in Henan Province (19A310024) and The National Natural Science Foundation of Henan Province (212300410074).

## Conflict of Interest

The authors declare that the research was conducted in the absence of any commercial or financial relationships that could be construed as a potential conflict of interest.

## Publisher’s Note

All claims expressed in this article are solely those of the authors and do not necessarily represent those of their affiliated organizations, or those of the publisher, the editors and the reviewers. Any product that may be evaluated in this article, or claim that may be made by its manufacturer, is not guaranteed or endorsed by the publisher.

## Abbreviations

Bv8, bombina variegata peptide 8; C4B-bp, C4B-binding proteins; CSCs, cancer stem cells; MDSCs, Myeloid-derived suppressor cells; MDSCs-Exos, exosomes released by MDSCs; G-MDSCs, granulocytic MDSCs; M-MDSCs, monocytic MDSCs; mRNAs, messenger RNAs; ncRNAs, noncoding RNAs; MIF, migration inhibition factor; PF-4, platelet factor-4; STAT, signal transducer and activator of transcription; TDEs, tumor-derived exosomes; PGE2, Prostaglandin E2; TGF-β1, transforming growth factor-β1; Th17, helper T cell 17; TME, tumor microenvironment; Tregs, regulatory T cells; NSCLC, in non-small cell lung cancer; G-CSF, granulocyte colony–stimulating factor; VEGF, vascular endothelial growth factor; Tsp1, thrombospondin1.
